# Glucagon as a Therapeutic Approach to Severe Hypoglycemia: After 100 Years, Is It Still the Antidote of Insulin?

**DOI:** 10.3390/biom11091281

**Published:** 2021-08-27

**Authors:** Francesca Porcellati, Stefania Di Mauro, Alessio Mazzieri, Alessandra Scamporrino, Agnese Filippello, Michelantonio De Fano, Carmine Giuseppe Fanelli, Francesco Purrello, Roberta Malaguarnera, Salvatore Piro

**Affiliations:** 1 Department of Medicine and Surgery, Perugia University School of Medicine, Via Gambuli 1, 06126 Perugia, Italy; francesca.porcellati@unipg.it (F.P.); alessiomazzieri90@gmail.com (A.M.); michelantonio.defano@libero.it (M.D.F.); carmine.fanelli@unipg.it (C.G.F.); 2 Department of Clinical and Experimental Medicine, Internal Medicine, Garibaldi-Nesima Hospital, University of Catania, 95122 Catania, Italy; 8stefaniadimauro6@gmail.com (S.D.M.); alessandraska@hotmail.com (A.S.); agnese.filippello@gmail.com (A.F.); fpurrell@unict.it (F.P.); salvatore.piro@unict.it (S.P.); 3 Faculty of Medicine and Surgery, University of Enna “Kore”, 94100 Enna, Italy

**Keywords:** diabetes, glucagon, hypoglycemia, therapy

## Abstract

Hypoglycemia represents a dark and tormented side of diabetes mellitus therapy. Patients treated with insulin or drug inducing hypoglycemia, consider hypoglycemia as a harmful element, which leads to their resistance and lack of acceptance of the pathology and relative therapies. Severe hypoglycemia, in itself, is a risk for patients and relatives. The possibility to have novel strategies and scientific knowledge concerning hypoglycemia could represent an enormous benefit. Novel available glucagon formulations, even now, allow clinicians to deal with hypoglycemia differently with respect to past years. Novel scientific evidence leads to advances concerning physiopathological mechanisms that regulated glycemic homeostasis. In this review, we will try to show some of the important aspects of this field.

## 1. Introduction

When insulin was introduced to the scientific world of diabetes in 1922, it immediately became clear that it was one of medicine’s most significant breakthroughs. Nevertheless, shortly after the discovery, Elliot Joslin wrote, “*insulin is not a cure for diabetes, but a potent preparation … for evil and for good*”. It might have seemed unusual for Joslin to describe insulin in this way, yet clearly, he was referring to the most common and feared side-effect of insulin therapy, hypoglycemia [1].

Moreover, in 1922 James Collip, while working with Frederick Banting on purifying insulin, in an attempt to measure insulin activity, identified one unit of insulin as the amount necessary to induce hypoglycemic seizures in a rabbit.

After 100 years, despite all the major advances in insulin therapy and blood glucose monitoring, hypoglycemia still remains *the dark side of insulin*, the major barrier preventing the full realization of its promise in diabetes management [2].

Hypoglycemia has severe clinical consequences. It is a risk factor for morbidity and mortality and heavily impacts the quality of life of people with diabetes and their caregivers.

Moreover, hypoglycemia may contribute greatly to increased health costs. Therefore, prevention and minimization of the risk and burden of hypoglycemia must be one of the main objectives in the treatment of diabetes. Hypoglycemia in people with diabetes is the consequence of treatment necessary to control hyperglycemia and prevent its long-term complications. However, pharmacological treatments for Type 2 Diabetes (T2DM) available on the market today, include a number of options. Some of these agents have a low risk of hypoglycemia and for this reason they are strongly recommended for patients for whom avoiding hypoglycemia is a clinical priority [3]. In such conditions, preferred pharmacological strategies include DPP-4 (Dipeptidyl Peptidase 4) inhibitors, GLP-1 (Glucagon-Like Peptide 1) receptor agonists, SGLT2 (Sodium-Glucose Cotransporter-2) inhibitors, and TZDs (Thiazolidinediones), while sulfonylureas and glinides should be avoided [3]. With regards to injectable treatments whenever possible, GLP-1 receptor agonists should be preferred to insulin. In Type 1 Diabetes (TD1M), insulin analogues, both short-acting and long-acting, should always be offered as an option, which more closely reproduce the physiological needs and translate into a lower risk of hypoglycemia given the specific pharmacokinetics and pharmacodynamics of both preparations [4,5,6,7,8]. Sensor technology is providing evidence of improving patients’ lives not only by attaining glycaemic control but also by reducing hypoglycemia. Therefore, the actual scenario in terms of therapeutic options, that may limit the risk of hypoglycemia in diabetes care, is doubtless far better than that of a few decades ago. However, mild hypoglycemia is still “a fact of life” for people with T1DM [9]. Severe episodes still represent a burden in terms of quality of life for people with diabetes, a diabetes management’s issue for healthcare providers, and a high cost for national healthcare systems.

This review first describes the epidemiological context of hypoglycemia and in particular severe hypoglycemia both in T1DM and T2DM, subsequently, it briefly focuses on its pathophysiology. The review further aims to illustrate ways and options to limit hypoglycemia in clinical practice, and the striking power of structured education programs. Last but not least, it shows the available treatments for severe hypoglycemia with a particular focus on the novel formulation of nasal glucagon.

## 2. The “Numbers” of Hypoglycemia

In people with diabetes, hypoglycemia is defined as a condition in which blood glucose level is lower than normal, thereby exposing the individual to potential harm [10,11]. Hypoglycemia in diabetes is usually defined by clinical criteria based on the severity of hypoglycemic events [12]. Non-severe hypoglycemia episodes (Level 1), are defined by plasma glucose concentrations between 54 and 70 mg/dL [3.0 and 3.9 mmol/L]. These non-severe events are generally characterized by a series of behavioral responses (symptoms), including the ingestion of carbohydrates, aimed at correcting of hypoglycemia and at preventing the further decrease in plasma glucose level and, as consequence, the risk of cognitive impairment. When glucose continues to decrease (Level 2), reaching levels that are less than 54 mg/dL [less of 3.0 mmol/L], or when patients need assistance from third parties (Level 3), we are in a condition of severe hypoglycemia (SH). When this situation occurs, assistance from another person to actively administer carbohydrates, glucagon, or to take other corrective measures is required to recover the clinical status, especially the functional brain failure of the affected individual [3]. Plasma glucose measurements may not be available during the hypoglycemic crisis, but the prompt neurological recovery after raising plasma glucose level is considered sufficient evidence that severe hypoglycemic episode took place.

In T1DM, the frequency of non-severe (mild) hypoglycemia is believed to be approximately 0.7–2 episodes/patient-week [13,14]. This estimate is most likely under-reported because main mild hypoglycemic episodes are often ignored, especially when occurring at night, or are treated by patients themselves. Therefore, detailed information on most of non-severe hypoglycemic events are not likely provided to doctors and healthcare visitors. However, mild hypoglycemia has relevant clinical implications and may result in serious acute consequences. In fact, recurrent episodes of mild/moderate hypoglycemia over a short time, may induce unawareness of hypoglycemia and counter-regulatory response, thereby predisposing to the risk of SH [1].

In contrast, much information is available on SH, as most episodes require the help of another person or hospital admission so that these hypoglycemic events are easily recognized by patients or their relatives/caregivers. Several scientific evidences indicate that rates of SH are higher in unselected populations than in those recruited in clinical trials where, almost always, people with risk factors for SH (i.e., hypoglycemia unawareness, previous episodes of SH, long duration of insulin therapy) are excluded [15]. Indeed, in an observational study in the UK, the rate of severe hypoglycemia ranged from 1.1 to 3.2 episodes/patient-year according to insulin treatment duration (<15 years and >15 years, respectively) [13]. This rate was substantially higher compared to that experienced by patients in the DCCT (Diabetes Control and Complications Trial) study [16]. Furthermore, according to a cross-sectional Danish-British multicenter survey, recruiting 1076 adult patients affected by T1DM, the incidence of SH was 1.3 episodes/patient-year. Differently, in a subgroup selected to be similar to the DCCT cohort, the rate of severe hypoglycemia was 0.35 episodes/patient-year A multicenter, observational, retrospective Italian study, reported an incidence of 0.49 severe hypoglycemic episodes/patient-year [17]. In both these studies, the distribution of severe hypoglycemia was highly skewed with fewer subjects accounting for most of the episodes [14,17]. More recent real-world evidence studies have shown the worrisome picture of an increased rate of severe hypoglycemia, in particular in younger adults, as compared to previous observations [18,19].

The recent SWITCH 1 randomized clinical trial, enrolling patients with at least one hypoglycemic risk factors, reported a frequency rate of hypoglycemia quite different. In particular, in this study, T1DM patients treated with insulin Degludec compared with those using Glargine U100, had a reduced risk of overall symptomatic hypoglycemia. In detail, the rate of severe episodes was 0.87/patient-year for insulin Degludec vs. 1.05 episodes/patient-year for insulin Glargine U100, far higher, for both arms, than that of the DCCT, in which only human insulin was available [20].

In T2DM patients, the incidence rate of SH ranges from 0 to 0.73 episodes/patient-year in people with T2DM [21,22], with several variables recognized to influence the episodes of hypoglycemia such as age, disease duration, intensification of glycemic control, use of insulin or sulfonylureas. However, several studies show that the rates of SH in T2DM, are lower than in T1DM [22]. This is not surprising as T2DM is characterized, at least in the early stages of the disease, by the presence of insulin resistance beside a persistent β-cell function. The maintenance of insulin secretion allows to decrease as blood glucose levels, and apparently, to maintain intact or even increased the counter-regulatory response. As disease progresses, in parallel with the loss of endogenous insulin secretion, T2DM resembles T1DM, and the risk of SH increases [13,17,21,23].

In conclusion, it is important to highlight that, in clinical practice, SH as well as non-severe episodes may be easily underreported and underestimated for several reasons. Many people with diabetes often do not recognize mild hypoglycemic events or, in the case of SH, they are often reluctant to discuss the episodes with their healthcare providers for a number of reasons (work issues, certifications and others). Therefore, it is important to directly query patients treated with an insulin secretagogue or with insulin about episodes of hypoglycemia at every medical examination [24].

## 3. Pathophysiology of Hypoglycemia and Its Consequences

Therapeutic hyperinsulinemia, either absolute or relative, as a result of exogenous insulin during insulin therapy or following insulin secretagogue therapy, is the initiating cause of hypoglycemia in diabetes. Unfortunately, it is not the only cause. As a matter of fact, in T1DM and advanced T2DM, there is a profound anatomic and functional derangement of intra-islet relationships between α and β cells, inducing the loss of cross-talking signals such as intra-islet insulin decrease during hypoglycemia. This event leads to the blunted or even absent response of glucagon to hypoglycemia, shortly after the onset of disease in T1DM [25,26,27]. Even in adolescents, glucagon response to hypoglycemia is blunted within the first year of diabetes diagnosis [28]. Loss of glucagon response to hypoglycemia in people with T1DM appears to be a “selective” decreased response of the α cells to glucose stimulus, as the response to non-glucose stimuli such as the amino acids arginine, alanine, and a mixture of amino acids is largely maintained [29]. Thus, there is a dual islet abnormality in diabetes, qualitatively common to both type 1 and type 2: The deficiency of insulin secretion in response to hyperglycemia, and the deficiency of glucagon secretion in response to the lowering of blood glucose by insulin. The latter defect increases the risk for hypoglycemia during insulin therapy or following insulin secretagogue treatment, and is one of the major factors of frailty in people with diabetes, especially when associated with reduced adrenaline response and with loss of symptoms to hypoglycemia (unawareness). Indeed, many patients with T1DM also suffer from reduced response of adrenaline, especially after recurrent hypoglycemia [30,31], and/or when the duration of T1DM is longer than 10–20 years [31]. The combined defects of absent glucagon secretion and reduced adrenaline response lead to the clinical syndrome of defective glucose counter-regulation that represents a strong determinant of future severe hypoglycemic episodes during intensive insulin therapy [32]. Antecedent hypoglycemia reduces autonomic and symptomatic responses to subsequent hypoglycemia in T2DM as it does in T1DM [30].

Hypoglycemia, depending on its severity, duration, frequency (acute vs. recurrent), and presence of comorbidities, can cause a plethora of severe consequences [11]. It can increase the risk of cardiovascular events [33,34], dementia [35], fractures [36], and overall mortality [37]. It also reduces the quality of life [38,39,40], and may generate fear of anti-hyperglycemic treatment, thus hampering efforts to achieve a good metabolic control.

Pathophysiological mechanisms linking hypoglycemia to some adverse effects and in particular to cardiovascular risk include, first, the release of catecholamines, which may induce increased myocardial contractility and workload, and cardiac output [34]. These responses can promote myocardial ischemia in patients with coronary vessel disease [41]. Increased sympatho-adrenal activation may further trigger a number of electrocardiographic abnormalities, such as a decrease in PR interval, moderate ST segment depression, and QTc prolongation [42,43]. The last could lead to a high risk of ventricular tachycardia and sudden death [43,44], in particular in the presence of hypokalemia, which may potentiate cardiac repolarization abnormalities. Along with the drive of the sympathoadrenal activation, several inflammatory markers including C-Reactive Protein (CRP), Interleukin 6 (IL-6), Interleukin 8 (IL-8), Tumor Necrosis Factor α (TNF-α), and endothelin-1, have been shown to be increased during hypoglycemia [45,46]. In addition, abnormalities in platelet function, activation of the fibrinolytic system, and endothelial dysfunction may play a role [47]. As a result, inflammation, platelet activation, procoagulant status, and endothelial dysfunction are closely interdependent [34,48].

**Figure 1:**
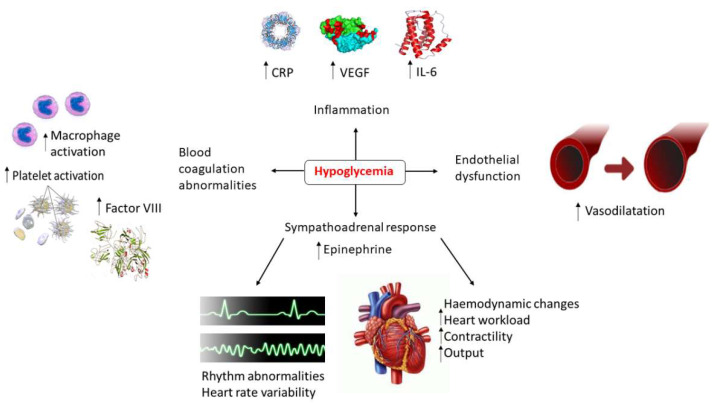
Potential mechanisms linking hypoglycemia to cardiovascular events.

Given this complex scenario (Figure 1), representing a sort of “tsunami” for the cardiovascular system, it is not surprising that the outcome of large randomized trials, looking at intensive glycemic control in T2DM, has shown no benefit to cardiovascular risk [49,50] or increased all-cause [51,52], and CV mortality [49,53]. It is beyond the scope of this review to discuss the possibility that iatrogenic hypoglycemia might be causally linked to the adverse consequences for the cardiovascular system, but we strongly agree with more recent interpretations, indicating hypoglycemia as a marker of vulnerability for adverse events [53,54].

## 4. How to Minimize Hypoglycemia in Diabetes Treatment

The first step to lower the risk of hypoglycemia during diabetes therapy is to carefully analyze each patient’s risk factors for iatrogenic hypoglycemia (Table 1). The most important and common of these are: a history of previous episodes of SH and a failure to recognize the impending hypoglycemic event. These two risk factors alone can increase the risk of SH by as much as six times.

Unfortunately, these two conditions are not always identifiable or discussed during medical examination, thus, hypoglycemic episodes are often underestimated by both physicians and patients. Although patients rarely report hypoglycemic events to healthcare providers, epidemiological studies show rates for SH in T1DM patients ranging from 70 to 159 events/100 person/years [15]. This estimate is higher than the incidence of SH reported in patients with T2DM, where the occurrence of severe hypoglycemic events is influenced by the type of glucose-lowering therapies so much so that it appears to be amplified by long-term intensive insulin treatment [15].

Hypoglycemia unawareness occurs in 17–36% of people with T1DM [55,56,57] and 6–8% of people with T2DM [58,59]. Indeed, many people have partial impairment of awareness of hypoglycemia and, as noted by Lawrence almost one century ago, even “the same patient may at one time experience, early premonitory symptoms and at another be quite unaware of an impending attack” [60]. Cryer has proposed the concept of “Hypoglycemia Associated Autonomic Failure (HAAF)”, in diabetes, speculating that previous episodes of hypoglycemia is the primary cause of both decrease of normal response to hypoglycemia and failure to recognize the hypoglycemic crisis. This sets up a vicious cycle in which hypoglycemia begets hypoglycemia [61]. HAAF, now known as “Cryer syndrome” [62], is largely considered a functional disorder distinct from classic diabetic autonomic neuropathy [63].

Because it has been reported a high prevalence of hypoglycemia unawareness even in persons with T1DM using Continuous Glucose Monitoring (CGM) [64], it is useful in clinical practice to quantify awareness of hypoglycemia using specific questionnaires as recently shown by observational studies both in T1DM and T2DM [64,65]. In these studies, three methods of assessing impaired awareness of hypoglycemia have been suggested, i.e., Clarke, Gold and Pedersen-Bjergaard methods.

Regarding to other risk factors for hypoglycemia, recent evidence, from real-world studies, both in T1DM and T2DM, no longer confirms the association between lower HbA1c levels and a higher risk for SH [19,66], supporting the concept that any HbA1c levels protect patients from severe hypoglycemic episodes. This statement gives more importance to some aspects of diabetes management, such as therapeutic options, glycemic targets and proper patient education, rather than the A1c value per se, in influencing the risk of hypoglycemia.

To better manage both blood glucose control and the prevention of hypoglycemic episodes, precipitating factors for hypoglycemia (Table 1) should largely be discussed with patients providing adequate knowledge [67]. This concept focuses attention on the relevance of implementing individualized and structured education programs in order to prevent hypoglycemia and restore awareness when it is lost [68,69]. Specific programs have been developed and incorporated in routine care to educate patients to recognize early hypoglycemic symptoms and situations that are risk-precipitating factors for hypoglycemia, thus improving glycemic control, quality of life, self-management and awareness of hypoglycemia while reducing the frequency of severe episodes [70,71].

Technology interventions include intermittent Flash Glucose Monitoring (FGM) and continuous glucose monitoring (CGM), which have been confirmed, in several observational studies, to decrease the rate of severe hypoglycemic episodes in insulin-treated patients [72,73,74,75,76]. Accordingly, Real Time Continuous Glucose Monitoring (rtCGM) can reduce the frequency of SH both in subjects with long-standing T1DM [77] and in those with hypoglycemia unawareness [78].

Notably, two above-mentioned papers have recently highlighted the role of technologies for improving hypoglycemia awareness [76,78]. The IN CONTROL study [76], enrolled a group of patients (29 in Multiple Daily Insulin Injections (MDI) and 23 in Continuous Subcutaneous Insulin Infusion (CSII)) with hypoglycemia unawareness and at high risk of severe hypoglycemic. This study showed that the number of severe hypoglycemic episodes decreased with rtCGM vs. Self-Monitoring of Blood Glucose (SMBG) (14 vs. 34 events, *p* = 0.03) with similar results in patients on either Continuous Subcutaneous Insulin Infusion (CSII) or MDI. The effectiveness of rtCGM in avoidance of hypoglycemia among high-risk individuals with T1DM in SMBG and treated with MDI was evaluated by Heinemann et al. [78]. This study was a 6-month, multicenter, open-label, parallel, randomized controlled trial performed in 12 diabetes practices in Germany (HypoDE, Hypoglycemia in Deutschland) with the aim to establish whether the incidence and the severity of hypoglycemia could be reduced through the use of rtCGM. The results indicated that between baseline and follow-up periods, mean hypoglycemic episodes per 28 days decreased from 10.8 to 3.5 in rtCGM patients whereas remained essentially unchanged (14.4 vs. 13.7) in participants using SMBG. The incidence of hypoglycemic events decreased by 72% in patients using rtCGM. Mean HbA1c values were unchanged in both groups. A limited number of severe hypoglycemic events was observed during the therapy and follow-up phases in the rtCGM group (24 episodes) compared to the control group (39 episodes). Using the Clarke questionnaire, the hypoglycemia unawareness score improved in both groups by approximately 40%, with no between-group differences [78].

Overall the data from these studies [76,77,78] support favourably the use of rtCGM in T1DM patients who are on CSII therapy but also in those treated with MDI. This latter group of diabetic persons represent the majority of type 1 treated subjects and for whom this technology may well help to prevent hypoglycemia.

Moreover, recently, either real-world studies or RCT (Randomized Controlled Trials) studies, have shown how, in T1DM subjects, CGM reduces the time spent in hypoglycaemia and the impaired awareness of hypoglycaemia more effectively than FGM, either in the blinded modality [79] or as rtCGM [80,81,82]. This supports that the switching from FGM to rtCGM represents a beneficial therapeutic option [81].

The use of CSII compared to MDI, gives some benefits in improving the awareness of hypoglycemic symptoms and the reduction of their severity [83]. Moreover, the insulin suspension features could further lower the risk of SH [84]. More innovative technologies, such as the hybrid closed loop (HCL) system, commonly referred to as an artificial pancreas, represent the most promising systems to achieve optimal glycemic control while minimizing the risk of hypoglycemia, and the occurrence of severe episodes [85,86,87,88,89]. Indeed, a recent study evaluating the effects of short-term use of an HCL system, although failed to demonstrate an improvement in hormonal counterregulatory hormonal responses, it showed higher hypoglycemia symptom scores during controlled hypoglycemia, better self-reported hypoglycemia awareness, and less time spent in hypoglycemia. These results indicate the potential benefits of an HCL system in people with impaired awareness of hypoglycemia [90].

Several studies have shown that proper education and structured programs are also important for introducing and guiding diabetic patients to the use of technology, from simpler to the more sophisticated devices. Indeed, patients properly educated, achieve a better avoidance hypoglycemic events because they better understand hypoglycemic symptoms and the way to self-manage them. This concept is supported by Little et al. which demonstrated that, in patients with hypoglycemia unawareness, it has been demonstrated how the impact of structured education and continuous support for patients might be more important than the use of CSII and CGM (vs. MDI and SMBG) [69]. This randomized 24-week clinical trial, with 2 × 2 factorial design, in adult subjects with T1DM and hypoglycemia unawareness, analyzed whether CSII versus MDI and the use of CGM compared to SMBG, with equal education, was able to improve awareness of hypoglycemia, measured according to the Gold and Clarke criteria, and the risk of severe hypoglycemia [69]. All enrolled patients received structured education/support and identical therapeutic goals, aimed at strictly avoiding hypoglycemia at 70 mg/dl. At the end of the 24-week treatment period, the frequency of hypoglycemia (< 3 mmol/L, 54 mg/dl) and hypoglycemic awareness, improved without changes in HbA1c. There was no substantial difference in results between CSII and MDI or between SMBG and CGM. These data indicate that hypoglycemia sensibility, severity and recurrence could be improved in long-lasting T1DM without worsening glycemic control. These results can be achieved with conventional MDI and SMBG regimes compared to CSII/rtCGM. Recently, the 2-year follow-up results of this study were published and substantially confirmed those observed at 24 weeks [91]. However, it should be noted that adherence to the use of the sensor was low [57%] and that the LGS (low glucose suspend) function was not activated in rtCGM users. Both of these elements may have underestimated the potential role of rtCGM in hypoglycemia prevention.

Despite different therapeutic options, technological strategies and the efforts of structured education programs, SH remains a major challenge in diabetes management.

## 5. Treatment of Severe Hypoglycemia (SH)

As already mentioned above, Severe Hypoglycemia (SH) is an acute and life-threatening event that could interfere with the subject’s ability to treat and take care of themselves. In this situation, assistance from another person is required to recover the clinical status of the affected individual. Despite progress in the field of therapies (i.e., second generation of insulin) or the field of technologies (i.e., flash glucose monitoring or continuous glucose monitoring), the occurrence of SH remains a difficult hurdle to overcome for both T1DM and T2DM [92]. For this reason, people with diabetes taking insulin, in particular T1DM subjects, should be informed about their hypoglycemic risk. Specific questionnaires should be used to evaluate this risk. Nevertheless, glucagon should be prescribed to all high-risk patients. This group of individuals should have the opportunity to carry and use very easily and quickly the glucagon delivery device in public places such as schools, gyms, restaurants, offices, etc. Relatives and colleagues should be instructed on how to use such devices and administer glucagon.

But why do we need to use glucagon to treat SH? Glucagon, a hormone produced by pancreatic alpha cells, regulates and prevents blood glucose-lowering and acts to quickly restore the normal levels of blood glucose. Under physiological conditions, glucagon regulates blood glucose levels in the fasting state. Insulin and glucagon control glucose homeostasis and prevent hyper and hypoglycemia, respectively. In diabetes patients, in particular T1DM, insulin therapy along with alpha cell-insensitivity to blood glucose levels, SH could represent a result of a defective balance between insulin and glucagon crosstalk, production, and action [93,94]. Since 1923, the year when glucagon was discovered, this hormone has represented a rescue strategy to treat and resolve hypoglycemic events in insulin-treated patients [95]. To date, glucagon injections are still the most important approach to recovery coma or to treat people unable to take care of themselves [96]. However, current injective devices containing glucagon are not so easy to use, prepare, and inject for patients, caregivers, and acquaintances. Often, caregivers and acquaintances wrongly think that glucagon injection requires medical assistance.

## 6. Current Strategies (Treatment) for SH

In this section of the article, we will illustrate the strategies to treat SH using different glucagon-based approaches.

Although hypoglycemia represents a key aspect of a diabetic patient’s life, especially for the insulin-treated patients, it is important to highlight that the risk to develop hypoglycemic events is not the same for each diabetic patient. Several aspects such as disease duration, remaining C-peptide levels, the specific type of used insulin, and employed diet could improve the identification of subjects at risk to develop SH. For instance, a very recent study [97] demonstrates that a preserved C peptide secretion is associated with less risk to develop hypoglycemic events and better glycaemic control in terms of glycaemic variability and time in range of glucose.

These new concepts increasingly allow characterizing the patient phenotype to minimize the risk to develop severe hypoglycemia.

To date, the available strategies for SH, especially in subjects unable to introduce carbohydrates orally, consist of intravenous dextrose and/or injectable glucagon administration. According to the guidelines of the international scientific societies, glucagon should be prescribed for all subjects at increased risk of developing SH and administered to subjects at risk of Level 2 Hypoglycemia (blood glucose less than 54 mg/dL or 3.0 mmol/L). Moreover, glucagon should be available in all emergencies [96]. For this purpose, ADA and ISPAD recommendations suggest that family members, caregivers, school personnel, personal trainers, and other persons that look after or interact with these diabetic subjects should be informed about where glucagon is stored, when, and how to administer it [96,98,99,100]. The above guidelines highlight that instructions concerning how glucagon should be administered are essentials for patients and caregivers. However, the guidelines recognize that the available formulation of injectable glucagon could represent an obstacle to correctly treat patients with SH or in a coma. For these reasons, every patient and family member should receive the correct information on these aspects. Diabetologists should periodically discuss this with patients and should ensure the correct delivery of information to patients and caregivers.

The GlucaGen HypoKit is from Novo Nordisk (Bagsvaerd, Denmark), GlucaGen^®^ Hypokit^®^, https://www.novo-pi.com/glucagenhypokit.pdf (accessed on 1 March 2021) and the Glucagon Emergency Kit is from Eli Lilly and Company (Indianapolis, IN, USA). The Glucagon Emergency Kit, http://www.lillyglucagon.com (accessed on 29 October 2018 and on 1 June 2021) is the injectable glucagon device currently available to treat SH. These two formulations can be used by subcutaneous or intramuscular injection [101]. However, since the two formulations contain powdered glucagon, both emergency kits must be reconstituted by a multi-step process before administration, according to the manufacturer’s instructions [102].

Similarly, GlucaGen^®^ Hypokit^®^ and Glucagon Emergency Kit are not user-friendly and the powder contained in both formulations needs to be dissolved before the injection. The “four steps” protocol required for powder reconstitution is not easy to carry out and glucagon could be ineffective if it is not correctly reconstituted and injected appropriately. In 2017, Yale et al. reported that the time to administer injectable glucagon is variable and sometimes it could be very long [103]. In this study, the authors report that none of all the instructed caregivers of unconscious patients simulating an SH event (simulation study) were able to prepare and administer glucagon. In particular, the authors reported that less than 60% of instructed caregivers were able to administer glucagon and more than 80% of acquaintances were not able to prepare it. Moreover, none of the acquaintances were able to deliver a full dose of injectable glucagon. In this study, the meantime needed to prepare glucagon, according to the four steps protocol, was more than 2 min. Surprisingly, although in simulation condition, some caregivers and acquaintances switched glucagon devices with insulin pens, wrongly injecting insulin.

These observations, reported by Yale et al., represent the real hard limitation to treat SH with injectable glucagon, in particular in the presence of unconscious patients.

For all these reasons and based on patient demands, an easier and safer treatment for SH is a clear unmet medical need.

## 7. New Strategies (Treatment) for SH

### 7.1. Nasal Glucagon

Although GlucaGen^®^ Hypokit^®^ and Glucagon Emergency Kit represent fundamental strategies to treat and care for SH, to date new opportunities are being developed and some of these have recently been approved in some countries for clinical use. In particular, Nasal glucagon (BAQSIMI from Ely Lilly and Company, Indianapolis, IN, USA) is a ready-to-use drug/device combination to treat SH in people with diabetes aged ≥4 years (Eli Lilly and Company, Baqsimi US Prescribing Information, http://pi.lilly.com/us/baqsimi-uspi.pdf (accessed on 30 July 2019 and on 1 August 2021).

This new formulation is a dry powder, which does not require preparation or reconstitution and is available in a single-use device. Using this approach, glucagon is rapidly and passively absorbed through the anterior nasal mucosa, without the need for inhalation [104]. This route of administration is suitable for a comatose person with profound neuroglycopenia and is more comfortable and preferable for patients and caregivers during SH events.

The possibility that glucagon could be administrated by intranasal delivery in a needle-free manner is an old dream beginning in 1982 and hypothesized by Pontiroli et al. [105,106]. In 1988, Freychet L. et al., using a solution with deoxycholic acid, administrated glucagon intranasally in nondiabetic subjects and T1DM affected individuals. In their reports, after 6 min of spray administration, both glucose and glucagon levels were increased. Moreover, they reported that hypoglycemic symptoms were relieved in about 7 min [107]. Although these studies were inspiring and enthusiastic, the chemical structure of glucagon (a rapidly degraded peptide hormone), the potential toxic risk linked to the solvents used to dissolve the powder, and the insensitivity of the industry to develop this approach, have delayed research in this field. Only in the last decade, nasal glucagon has become a new reality.

To date, several studies regarding nasal glucagon administration have been conducted and include Pharmacodynamics (PD) and Pharmacokinetics (PK) studies [108], preclinical animal studies [109], adult and pediatric efficacy and safety studies [103,104,110] studies performed on T1DM and adult diabetic patients [110,111], in insulin-treated T2DM patients [112] or in a Real World setting or in particular conditions such as nasal congestion or a cold [108,111]. In all the above studies, no major side effects have been reported following nasal glucagon therapy. Nausea, headache, vomiting, and transient nasal congestion have been reported as minor side effects. It is important to note that nausea and vomiting were not so different between classic injected glucagon and nasal glucagon administration. A meta-analysis recently reported that nasal glucagon is equally effective to resolve hypoglycemic events and equally safe compared to injectable glucagon [113]. Although most of the studies were conducted in DMT1 patients, we report a study conducted in insulin-treated DMT2 patients [112]. In this phase 3 study, performed in Japanese patients, nasal glucagon was non-inferior to intramuscular glucagon for successful insulin-induced hypoglycemia.

Moreover, a cost-offset and budget impact analysis conducted by Pöhlmann et al. in the USA, indicates that nasal glucagon could have the potential to improve hypoglycemia emergency care and reduce SH-related treatment costs [114].

Due to the safety of these studies, nasal glucagon has now been approved as therapy for SH in most countries.

### 7.2. Liquid Glucagon

In addition to nasal glucagon, liquid glucagon is a new opportunity, recently approved in some countries for the treatment of SH in diabetic patients. This ready to use formulation is contained in a pre-filled syringe or a pen without a visible needle that requires only administration without any need of reconstitution (Xeris Pharmaceuticals, GVOKE US Prescribing Information, 2019 https://www.accessdata.fda.gov/drugsatfda_docs/label/2019/212097s000lbl.pdf, (accessed on 7 September 2019 and on 27 August 2021).

The device, in some countries called “Gvoke”, contains a room temperature, a liquid-stable form of glucagon, which goes through auto-injection when pressed against the body. Pre-filled rescue pens without a visible needle or pre-filled syringes are formulated for use in patients over the age of two and can be administered by caregivers in hypoglycemic emergencies.

Pens or syringes contain 0.5 or 1.0 mg of glucagon, which are the recommended doses for pediatric or adults use, respectively. One feature of this new formulation is the possibility to be stored at room temperature. In the USA, on 10 September 2019, the FDA approved and introduced this new formulation and its device as an emergency glucagon rescue treatment for SH [11,115].

Preclinical and clinical studies were conducted to establish the efficacy and safety of this formulation and to evaluate whether this device is easily usable [115]. In particular, so far PD and PK studies, preclinical animal studies, adult and pediatric efficacy and safety studies, and studies concerning the practicality of the device are available [11,115].

Two phase 3, randomized, controlled, double-blind, crossover clinical trials were conducted on T1DM adults in order to compare a subcutaneous 1 mg dose of glucagon (administered using a standard device) and liquid glucagon [11]. In both of these phase 3 studies, the liquid glucagon rescue pen was more effective compared to the conventional injectable glucagon emergency kit. Moreover, although the incidence of all adverse events was comparable between the two treatments, the liquid glucagon rescue pen was more appreciated than injectable glucagon [11,115]. The most common side effects reported in these trials were nausea, headache, vomiting or swelling at the injection site. To date, no major side effects have been reported in available clinical trials or clinical case reports. Moreover, to date, head-to-head studies comparing liquid glucagon to nasal glucagon are not available.

Liquid glucagon is available in some countries in a single-use pre-filled syringe or a needle-free device.

### 7.3. Other, Ongoing or Discontinued, New Glucagon Formulations

Naïve glucagon, coming mainly from pancreatic alpha cells, is a peptide hormone rapidly degraded in vivo and unstable in aqueous solution. Under this last condition, synthetic glucagon undergoes too rapid degradation leading to a loss of its activity. Due to the above limitation, specific approaches have been proposed in an attempt to extend glucagon stability in aqueous solution. Analogs of human glucagon with specific amino acid substitutions have been proposed. In particular, a glucagon formulation containing a substitution in amino acids 7 and 29 have been used. In this formulation, called Dasiglucagon (or ZP4207), glucagon has been proved to be stable in aqueous solution. The company producing this modified peptide, as declared on its website page, is developing this formulation to be used through pens or pumps. Despite this encouraging news, data regarding phase 3 studies in diabetic patients are still missing, especially those regarding immunogenicity due to amino acid sequence change.

Another possible approach is the opportunity to use a solvent that could avoid peptide degradation. Some industries have been tried to use polar solvents, such as the DiMethyl Sulfoxide (DMSO), which is generally used in other approved injectable products. Phase 2 trials indicate that these formulations could be delivered through pens, pumps or subcutaneous devices [11,115]. To date, also for this approach, data in diabetic patients are needed.

Recently, BIOD-961 has been developed and proposed. This new formulation consists of lyophilized glucagon in an auto-reconstitution device, which does not require external reconstitution. Few available data regarding BIOD-961, indicate a PD/PK profile similar to injectable glucagon [116,117]. However, no data are available in clinical setting or in humans.

Some other industries are developing a Biochaperone approach using native glucagon in an aqueous solution at neutral pH. Biochaperones are polymers or oligomers or organic compounds. Using this technology, it is possible to form aggregates, polymers, and complexes with proteins in order to protect glucagon from degradation. Animal Studies show that Biochaperones have PK/PD profiles similar to naïve glucagon [116]. However, further studies are needed to establish long-term safety and efficacy of these new formulations.

Other technologies have been described but some of them have been discontinued. For example, a transdermal approach, called ZP glucagon, was initially introduced as a new and promising strategy. However, studies regarding its safety and efficacy were disappointing and for this reason, the experimental research in this field was discontinued [11,115,116]. A similar approach using subcutaneous delivery, called SAR438544, was proposed by different industries, SAR438544 is derived from the Exendine-4 sequence. Due to the disappointing PD/PK results obtained by recent studies, this approach was interrupted and also this program is no longer under development [116].

## 8. Why Do We Have to Use Novel Routes of Administration if We Can Use Injective Glucagon?

The possibility to use novel preparations based on glucagon raised new questions. If glucagon is used to solve an acute problem (hypoglycemic event or Severe Hypoglycemia), often in an emergency, is the administration route important? Why should we take into account administering it through other routes than those currently used: Intravenous (i.v.) or Intramuscular (i.m.) administration? Certainly, at first sight, we could believe that these kinds of scientific advances add little to what is already known. However, in our opinion, the available data regarding safety, tolerability, usability, and patient preferences make us concerned [111,118]. Medical and scientific research points of view, do not always coincide with the patient’s desires and expectations. We should always take into account this aspect. Furthermore, it is important to highlight that these novel strategies, besides being more appreciated by patients, often offer novel and better therapeutic responses. We report, for instance, the study of Suico et al., where it has been demonstrated that the use of nasal glucagon was able to solve a hypoglycemic crisis in a similar time as an i.m. glucagon injection but with a more physiological hyperglycemic effect (less marked and longer lasting) [119].

This aspect certainly represents an advantage in addressing post hypoglycemic crisis needs. The exaggerated rise in glycemia after glucagon administration often induces patient guilt that could discourage therapy. Glucagon kinetics offer novel intervention possibilities, maintaining the same levels of safety and efficacy with respect to i.m. glucagon. Further information concerning these topics will certainly help our understanding of these novel therapeutic options.

## 9. Glucagon: Insulin Antidote or Main Driver of Diabetes Homeostasis?

Over the last 20 years, we have reconsidered several aspects of glycemic homeostasis that we used to consider from an insulin-centric point of view. Diabetes hyperglycemia is regulated by insulin level and insulin action in peripherical tissues. Glucagon, mainly produced by alpha-pancreatic cells, although it is an important approach to handle hypoglycemic events, has hardly been considered a protagonist of diabetes physiopathology. On the contrary, Unger RH, for a long time has supported the importance of this hormone for diabetes onset [120,121].

**Figure 2:**
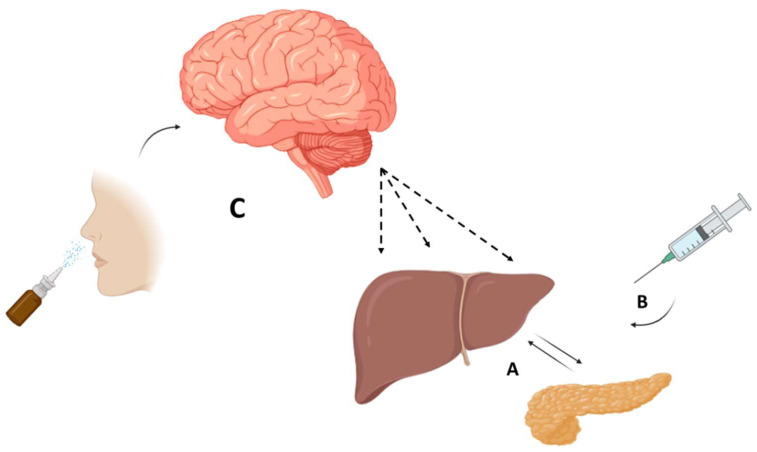
Hypothetical pathway of glucagon action/modulation on liver. (**A**) In physiological conditions, blood glucose regulates insulin and glucagon secretion. The equilibrium of these two hormones controls hepatic glucose release. (**B**) When glucagon is intramuscularly injected, it acts mainly at the hepatic level resulting in the activation of glucose release through glycogen lysis and gluconeogenesis. (**C**) Nasal glucagon administration could induce actions at the brain levels. In this way, novel potential actions at the hepatic level could regulate hepatic glucose release.

Unger’s hypothesis, experiments, and speculations deserve a detailed focused study in order to rewrite whole chapters of the diabetes medical field. By studying glucagon, indeed, it is possible to understand organ functions and dysfunctions that determine glycemic homeostasis. Gastrointestinal hormones, especially GLP-1, share glucagon roles and destinies and contribute to glycemic level regulation [122,123]. When glucagon is inappropriately high or is inadequately produced in response to glucose levels there are metabolic imbalances that go beyond glycemic levels, given by, for instance, the delayed response to hypoglycemia or the excessive hepatic glucose release after glucagon stimulus. These aspects, as widely known, are often observed in diabetic patients and they go beyond simple hypoglycemia [124]. Severe hypoglycemia, for instance, is an index of glucagon disequilibrium and impairment of alpha pancreatic cells. Subjects who develop severe hypoglycemic crises often have underlying alpha cell insensitivity to hypoglycemia and probably these cells respond inadequately to secretory or inhibitor stimuli. This is the reason why severe hypoglycemic events are recurring in diabetic subjects with long disease duration with respect to recently diagnosed diabetic patients. The knowledge of particular aspects concerning glucagon history and its origin could allow us to think about not only the causes determining glycemic increase but also to understand the contexts in which glycemia could be inappropriately low. Studies conducted by Lewis’s research group, in which nasal glucagon was used, suggest novel scenarios regarding the potential use of glucagon through the nose in clinical practice [125]. In these experiments, it is innovative that glucagon administration through the nose induces a more physiological and less pronounced response in terms of hepatic glucose release. On the one hand, this aspect could represent an advantage, on the other hand, for physiopathology researchers this represents an unexplored and novel aspect in the physiopathology of diabetes. We could hypothesize that the downstream effect of nasal glucagon administration in regulating hepatic glucose release could be influenced by the regulation mediated by the central nervous system rather than the simple direct effect on the liver (Figure 2). Nasal administration could stimulate both endocrine and nervous systems and this aspect could result in a more advantageous SH recovery.

This effect, although it still needs to be completely understood, could pave the way to novel aspects of body glucagon action. In this way, as Unger supported for his whole life, glucagon could regulate glycaemia level but it could be much more, it could represent the real cause of diabetes, overcoming the role of insulin and its deficiency. In this case, for example, the hypoglycemic action of glucagon, mediated by the central nervous system action, could demonstrate a central role not only at the hepatic level.

What we have reported above is absolutely consistent with Unger’s scientific production. We would like to think that Unger had the correct interpretation of diabetes pathogenesis and that taking into account these cultural advances concerning glucagon, we can soon arrive at the key to understanding diabetes.

## 10. Conclusions

After 100 years from the discovery of insulin, is it still the time of insulin? Of course, yes, it is. However, after one century from the discovery of insulin and 98 years from the discovery of glucagon, we should recognize that also this latter hormone plays a critical role in diabetes pathophysiology. If there still exists a “dark side of insulin” maybe it is due to the fact that glucagon has often been overlooked in our equations containing insulin and glycemia. Glucagon solves hypoglycemia but regulates many more things than we can imagine. Investing in glucagon, alpha pancreatic cells, and what is related to these, could allow us to better illuminate the obscure aspects that affect the lives of our diabetic patients.

## Figures and Tables

**Table 1:** Risk and precipitating factors for hypoglycemia.

Risk Factors	Precipitating Factors
A history of severe hypoglycemia	Insulin overdose in relation to CHO intake
Hypoglycemia unawareness	Delayed or missed meal
Stringent glycemic control	Prolonged exercise without control of glucose levels w/o insulin or food adjustments
Disease duration	Prolonged fasting Prolonged fasting in the presence of long-acting insulin analogs therapies
Increasing duration of insulin therapy (T2DM)	Alcohol ingestion
Extremes of age (very young and very old)	Factors influencing s.c. insulin absorption
Diabetic neuropathy	Intercurrent acute illnesses
Renal or hepatic impairment	
Neoplasms	
Number of drugs other than antidiabetic agents	
Social isolation	
Lack of proper patients education	

## Data Availability

Not applicable.
